# Call to Action: Investigating Interaction Delay in Smartphone Notifications

**DOI:** 10.3390/s24082612

**Published:** 2024-04-19

**Authors:** Michael Stach, Lena Mulansky, Manfred Reichert, Rüdiger Pryss, Felix Beierle

**Affiliations:** 1 Institute of Clinical Epidemiology and Biometry, University of Würzburg, Josef-Schneider-Straße 2, 97080 Würzburg, Germany; lena.mulansky@uni-wuerzburg.de (L.M.); ruediger.pryss@uni-wuerzburg.de (R.P.); 2 Institute for Medical Data Sciences, University Hospital Würzburg, Josef-Schneider-Straße 2, 97080 Würzburg, Germany; 3 Institute of Databases and Information Systems, Ulm University, James-Franck-Ring, 89081 Ulm, Germany; manfred.reichert@uni-ulm.de; 4 National Institute of Informatics, Tokyo 101-8430, Japan; felix.beierle@uni-wuerzburg.de

**Keywords:** notification, call to action, response time, HCI, mobile computing

## Abstract

Notifications are an essential part of the user experience on smart mobile devices. While some apps have to notify users immediately after an event occurs, others can schedule notifications strategically to notify them only on opportune moments. This tailoring allows apps to shorten the users’ interaction delay. In this paper, we present the results of a comprehensive study that identified the factors that influence users’ interaction delay to their smartphone notifications. We analyzed almost 10 million notifications collected in-the-wild from 922 users and computed their response times with regard to their demographics, their Big Five personality trait scores and the device’s charging state. Depending on the app category, the following tendencies can be identified over the course of the day: Most notifications were logged in late morning and late afternoon. This number decreases in the evening, between 8 p.m. and 11 p.m., and at the same time exhibits the lowest average interaction delays at daytime. We also found that the user’s sex and age is significantly associated with the response time. Based on the results of our study, we encourage developers to incorporate more information on the user and the executing device in their notification strategy to notify users more effectively.

## 1. Introduction

Notifications are designed to signal the user and to attract the attention the latter. They either remind users, provide them with information, or prompt them to take action. This feature was incorporated into operating systems (OS) before the advent of the smartphone and has been part of human–computer interaction design for decades. In contrast to the desktop PC realm, notifications in mobile computing are a much more integral part of the user experience, as the smartphone is usually within the user’s reach much more often than a traditional desktop PC [1]. Thus, the installed mobile applications can persuade users to interact with the smartphone virtually every minute of the day and therefore increase engagement. In [2], for example, the introduction of a notification mechanism, which served as a call to action, quintupled the frequency of data collection in their health service. A notification as a call to action is particularly effective if the notification offers action buttons to facilitate the user’s interaction [3].

However, for some applications, this pervasive capability to attract the users’ attention has become a prerequisite for reliable operation. Apps, for example, where a call to action is an integral part of the functionality (e.g., the app category of instant messaging) require the operating system to provide a reliable way to notify users in a timely manner [4]. In addition, less prominent app categories, such as health, also rely on a robust notification mechanism [5]. Daily life research methods such as Ecological Momentary Assessment or Experience Sampling, for example, are often used in studies in the healthcare domain and heavily utilize notifications to signal study participants [6,7,8]. Sometimes their sampling design is limited to only a small period of time for user interaction [9].

In recent years, the reliability of the notification management implementations of apps not using proprietary services offered by OS vendors is challenged by newly introduced battery optimization features in the Android OS versions 6, 7, 8, and 9. More specifically, the OS uses the interaction history of mobile applications to intelligently manage resources (i.e., optimizing energy consumption). These features especially compromise the execution of infrequently used apps, causing them to no longer operate as intended by the developer [10].

In addition to the technical requirements for a reliable notification mechanism, often implemented using proprietary solutions from the OS provider (i.e., Firebase Cloud Messaging and Apple Push Notification Service), a more in-depth analysis of user behavior is also beneficial to improve the effectiveness of notification mechanisms. For example, studies on notifications have shown that users prioritize app notifications differently, which influences how they interact with the notification [4,11,12,13]. At the same time, the number of notifications can vary greatly per mobile application [14,15] and depend on the hour of the day. Investigating these differences in a large scale can help to improve future notification management by designing smarter scheduling mechanisms. The latter, in turn, has the potential to improve the user experience of an app by incorporating these usage patterns and helps to minimize notification intrusiveness by optimizing notification delivery windows [16]. In the case of health apps, user data can help tailor notifications to the user’s individual health routines, increasing the perceived value of the app and the likelihood of future user engagement [17]. Optimizing user engagement and preventing notification fatigue can improve overall app effectiveness by increasing user adherence [18].

This study pursued the following several objectives: Firstly, it aims to quantify user behavior in terms of notification prioritization, response times, and the impact of notification volume. This analysis will include variations between app categories and time of day. Secondly, it aims to investigate how user demographics (such as age and gender), personality traits, app category, and device charge level influence user response time to a notification. By understanding user behavior patterns and preferred interaction times, the study aims to contribute to the development of intelligent notification scheduling systems. These systems could optimize notification delivery windows to minimize intrusiveness and improve the user experience, leading to improved app effectiveness and user engagement. This is particularly important in the area of health apps where user adherence is crucial.

In this work, we provide such insights into the interaction of smartphone users and their apps. Based on the TYDR dataset [19,20,21], we analyzed the interaction with smartphone notifications together with other user- and device-related data to identify factors that influence the users’ smartphone interaction. More specifically, the core contributions of this work are as follows:

- A detailed exploration of in-the-wild smartphone notifications of a large dataset;
- A comprehensive analysis of temporal differences in user interactions to determine preferred times for notification scheduling;
- The role of the users’ demographics and personalty traits for notification scheduling;
- The impact of the app’s category and the devices charging state on response times to smartphone notifications.

The results shall support developers to design more reliable notification services and to improve the alignment of notification schedules to the common users’ interaction patterns by incorporating additional information.

The article is structured as follows: Section 2 provides background information on smartphone notifications, with a focus on the notification mechanisms offered by the Android operating system. Section 3 describes the methods used to collect, process, partition, and analyze the data. In Section 4, we present the results of our study, and in Section 5, we discuss their implications. Finally, in Section 6, we conclude our work and provide an outlook for future research.

## 2. Related Work

There are several works that investigated the user’s perception of and interaction with smartphone notifications. Since various mechanisms in current smartphone OSs can interfere with the notification creation process, we also provide a brief description of the issues as well as technical background knowledge in this section.

### 2.1. Notification Interaction

In several works, smartphone users showed that their behavior regarding the interaction with smartphone notifications is complex and also differs in many regards. Ref. [1], for example, found an association between call durations and the number of notifications per day as well as the level of extraversion. Other personality traits, like neuroticism, are also positively related to smartphone usage [22]. In addition to personality traits, ref. [23] examined how people use smartphones depending on their age and gender. According to the study, women tend to use their phones longer than men, with an average daily usage time of 167 min compared to 154 min for men. Additionally, women tend to spend more time using communication and social apps, while men spend more time playing games. These findings suggest that age and gender are significant factors in determining how individuals use their smartphones. A classification of users in terms of notification management was also shown in [14]. Interestingly, this is also observed in the opposite direction: user behavior can be influenced by a call to action in the form of a smartphone notification [24]. This shows the significance of a comprehensive understanding of how users interact with notifications and what influences these interactions.

In their study on notifications, Pielot et al. [15] found that participants typically viewed notifications within a few minutes, but also perceived them as disruptive. Additionally, participants reported feeling stressed and overwhelmed by the number of notifications received, particularly from messaging apps. Weber et al. [25], in turn, investigated how users deal with interruptions from mobile notifications by manually deferring them. Previous research has focused on delivering notifications at convenient times to avoid interrupting users. This study examined a different approach: an app called “NHistory” that allows users to ’snooze’ notifications for a specific time or until a specific time. Users typically snooze notifications for short periods of time, usually no longer than two days. The most frequently snoozed notifications include messages, calendar notifications, social media, and email notifications. To identify opportune moments for user interaction, ref. [26] developed a machine learning model. The model significantly outperformed the baseline model and predicted user engagement with recommended content 66.6% more accurately. It uses phone usage patterns, communication activity, and context such as location to make these predictions. Although this approach theoretically enhances user engagement with mobile notifications by sending them at optimal times, the study only examined engagement with a specific content format. It is unclear whether the model can be generalized to other notification types or content.

To shed light on the notification ecosystem, ref. [4] conducted, to the best of our knowledge, the largest study (regarding the amount of participants and notifications) in the literature. Even though the study is from 2014, the paper provides a broad look at how people interact with notifications, as they collected almost 200 million notifications of more than 40,000 users. In their study, they found that the amount of notification a user receives differs for each app category. WhatsApp, for example, was particularly prevalent in their data, leading the authors to suspect that it was the most widely used and most common app of their study participants. The second most common app category was e-mail, followed by SMS. Participants of this study, furthermore, stated that notifications were important for them, especially when the notification is related to communication. For these reasons, the authors assume that communication remains one of the most important functions of smartphones, despite the large variety of apps and usage possibilities. However, the study differs from the TYDR project in its technical structure: Ref. [4] created both a mobile and a desktop application in the form of a web browser plugin for Google Chrome and Mozilla Firefox. The desktop application served as an incentive for study participants to view notifications from their smartphone on their PC. This could have an impact on the study’s participant selection, as the use case mainly targets users of both mobile and desktop devices. Another difference between [4] and our work is the availability of user demographic data in TYDR. In this work, we show that additional information on the app user can make an important contribution to understanding user interactions with notifications.

In [27], the authors studied interruptions of smartphone users caused by notifications. Following a comparable in-the-wild data collection approach to the TYDR app, the authors used Mobile Crowdsensing to log data in the background and Experience Sampling Methods to collect additional data (e.g., Big Five personality traits) using questionnaires. They found that the presentation, alert type, sender–recipient relationship and characteristics of the task influence the user interaction. Furthermore, they found a significant correlation between a notification’s seen time and the user’s extroversion and neuroticism [27]. In contrast to our work, they focused not on the temporal delay of an interaction, but on the users’ receptivity to notifications. Furthermore, their work does not differentiate results by app category. The elaborate assessment of notifications is perhaps the reason that only a fraction of people participated in the study (*n* = 20) compared to the TYDR study (*n* = 922).

In another work, ref. [11] analyzed in their study a larger dataset containing roughly 800,000 notifications from 278 users. They also found that instant messaging notifications are interacted with the fastest. Furthermore, they showed that a surprisingly large number of notifications were received while the user is actively using the phone. When looking at the restrictions described in Section 2.2, this directly influences the timely creation of the notification. However, in contrast to our work, they focused on the daily number of notifications instead of a per-hour evaluation and, furthermore, calculated conversion rates for the notifications (i.e., percentage of interaction-triggering notifications). Again, the app category “messages” was the most interesting from the users’ perspective, since this category showed the highest conversion rate.

Table 1 compares the research in this section with the study presented in this paper, highlighting the different parameters analyzed in each study. Our study included more parameters in the overall analysis, as we included demographic information, personality traits, app-related data, and battery status, while the other studies used fewer parameters for their analysis.

### 2.2. Technical Background

Since notifications constitute an important pillar of modern applications and their pursuit for attention, current mobile OSs like Android have introduced complex features for notification management. From a technical perspective, applications use OS interfaces to instruct the OS to either set up background services that create notifications depending on incoming messages from a remote entity (remote push notifications), or to schedule the creation of notifications at a specific time (local push notifications).

However, changes to the behavior of OS interfaces and newly introduced restrictions on the execution of background services can be error-prone for all those apps that have not yet been updated or tested for a new OS version. For example, ref. [28] describe in their work the behavioral change in their mobile crowdsensing app with different Android versions. They further describe that the background data collection was affected by the introduction of various battery optimization features. Problems with these OS-related restrictions regarding background services were also reported in [14]. Vendor-specific OS adjustments like a battery optimization feature had such an impact on the data collection that those datasets had to be excluded from the data analysis. To avoid such issue in the TYDR app, the data collection tasks have been implemented as a foreground service (see Section 3.1).

Since there is, according to [29], a certain amount of unawareness in the developer community about OS’ internal mechanisms that affect the execution of apps and their background services, we briefly describe the following two prominent methods to execute background tasks on Android as well as limitations posed by the OS:

The background service offers the possibility to continuously run application code in the background for a longer period of time and without the user’s awareness. This is, for example, especially useful when notifications or messages need to be received from and processed by the server at all times and without the app’s prior knowledge. Since background services are controlled by the app and thus represent potential battery consumers from the OS’ perspective as well as possibly accessing sensitive information without the user’s knowledge, they are subject to some restrictions in the latest Android versions. In addition, background services can be stopped by the OS at any time, which is why a reliable execution cannot be guaranteed for the entire operating time of the smartphone.

The second way to run application code in the background is through the alarm-based approach. Via the alarm interface, the Android framework provides a way to schedule tasks in advance and execute them at a later point in time. The OS then takes over the management of the tasks, which is why this variant is considered particularly energy-saving. Depending on the intended use, however, this can affect the timing of the tasks since alarms are extremely dependent on the battery optimizations integrated in the OS like Doze or App Standby [30,31,32,33].

The most significant restriction of the aforementioned Doze mode is that apps can perform background tasks only in a periodically offered maintenance window. This restricts apps from precisely create notifications without including mechanisms that also work on Doze mode (e.g., proprietary services that are excluded from OS restrictions, such as Firebase Cloud Messaging). As soon as the user activates the device or charges the device, Doze mode is terminated and therefore all restrictions are suspended. Both, Doze and App Standby have large impact on applications [10] that are not designed for optimal operation under this execution environment. This may also be evident in the creation of notifications, as, for example, notifications are not created until the next maintenance window and thus will be displayed delayed.

## 3. Methodology

We analyzed the data collected with the app TYDR (Track Your Daily Routine) [19,20,21] to better understand differences in the usage behavior of app users and support future apps in their effort for strategic notification scheduling. TYDR (Google Play Store entry: https://play.google.com/store/apps/details?id=de.dynamic_project.tydr (accessed on 23 February 2024)) is an app developed for research purposes for Android smartphones that used the mobile crowdsensing methodology to collect sensor data and smartphone usage statistics as well as pose questionnaires on the personality of the users. From a user’s perspective, TYDR shows aggregate statistics about the user’s smartphone use. From our research’s perspective, we collected these data in order to analyze smartphone usage behavior. When opening the TYDR app, the user can choose which data he or she is willing to let the app track and display statistics about.

For this work, we used a subset of the TYDR dataset, containing notification metadata and battery data. In addition, we used the gathered information about the users’ devices as well as the responses to a demographic and a personality traits questionnaire. The data were collected between October 2018 and October 2020.

### 3.1. Data Collection

From the user’s perspective, in order to let TYDR collect notification metadata, he/she has to allow TYDR to access notification data, and, in turn, see aggregate statistics about how many notifications were triggered by which app. Once the permission is given, we store metadata for each notification in a local database, and periodically upload it to our backend. For more details on the implementation and privacy aspects, please refer to [19,20,21].

To track the interactions of users with their smartphones, TYDR implemented a so-called notification listener that logs all notification metadata, e.g., app name, timestamp of the appearance and disappearance of the notification. The notification listener was implemented as a foreground service instead of a background service with a permanent notification to indicate the ongoing data collection. This was necessary in order to prevent the OS from stopping the TYDR app listeners (cf. Section 2.2) and thus not missing any app interaction. In order to preserve the users’ privacy, all fields containing private data (e.g., title or content of the notification) were only recorded in hashed form.

The battery-related data were gathered using the BatteryManager interface of the Android OS. The BatteryManager uses broadcasts to signal any changes in the batteries’ charging state. In addition to the charging state, the current battery level was also logged. Additional data regarding the executing environment (i.e., smartphone model name), demographic information of the user (e.g., sex, age), or other user-related information were collected either using programmable interfaces that were provided by the OS or by asking the user to fill out questionnaires.

### 3.2. Data Preprocessing

We deduplicated the battery and notification data, since the TYDR app logs data on every status change (e.g., due to an app-triggered update). To minimize anomalies in the Android-related data, we searched the smartphone dataset for smartphone updates. To be more precise, we excluded data from users who updated their operating system while using the TYDR app to ensure user-level data integrity. With the now cleaned data, we were able to calculate the following metric:

Interaction Delay (IDL) describes the temporal difference between the notification being displayed and the notification being removed from the notification bar (by clicking or dismissing).

The IDL was calculated as an integer to the second. We removed all records whose IDL was not greater than zero, as such short interaction times likely indicate automatic removal rather than human–computer interaction.

We then merged the battery data with the notification data based on timestamps and user identifier. Notification data and battery data might not be recorded at the same time. In the merging process, to enrich the notification data, we allowed for a time difference of 10 min between notification timestamp and battery recording. We expect the battery level not to change significantly in such a short period of time and allow for significantly more battery level annotation.

Since our analysis is dedicated to the temporal dynamics of notification creation and interaction, we have to distinguish between fixed notifications and notifications as a call to action. In order to disregard fixed notifications (and faulty records), we set an upper bound for the calculated IDL of one day. This limit excludes only a minimal number of 6496 records (0.06%) from the analysis and has no significant impact on the calculations of the analysis.

Each record can be associated with a device. Through this relationship, the records were enriched with additional information about the device (e.g., Android version and device model ID). To group the apps, we defined a list of 15 categories and manually sorted each app into one of the following categories: *Health*, *Finance*, *Outdoor*, *Shopping*, *Educational*, *Self-Organization*, *File Processing*, *Gaming*, *News & Entertainment*, *Social Media*, *Messaging*, *System-/OS-related*, *Misc*, *Warning*, and *less than 10 unique users*.

Finally, we removed all records that were either not complete (i.e., have attributes without information) or in the app categories *Misc*, *File Processing*, and *less than 10 unique users*. Doing so, we ensured on the one hand to only compare datasets containing a minimum amount of users, and on the other to exclude apps that are neither using notification as reminders nor as a call to action. For example, most notifications in the app category *File Processing* originate from cloud and office apps that use notifications to communicate the status of an ongoing task (e.g., file upload). We excluded these records because our purpose in this paper is to analyze human–computer interactions.

In addition to the records described above, we included the time of the day in hours (12 a.m.–11 p.m.) when the notification appeared, the battery level at the moment of the notification appearance, as well as whether the smartphone was charging at the moment (yes/no).

### 3.3. Datasets

After the data processing, the dataset contained 9,894,656 notifications from 922 unique users. In the following, this dataset is called DS1. In addition, we formed a second dataset (DS2) containing only data from users who have also filled out a demographic questionnaire (58%) and a third dataset (DS3) with users that filled out a demographic as well as a Big Five personality traits questionnaire (45%). In Table 2, we give an overview about the datasets. DS2 contains the users that filled out a demographic questionnaire. Overall, 84% of users are male (n=451) and 16% are female (n=86). The mean age of users is 35.2 years with a standard deviation of 10.6 years. DS3 contains the users that additionally filled out a Big Five personality trait questionnaire (BFI-2, [34,35]). The measured Big Five personality traits of TYDR user are comparable to those of the population average with only minor differences [21].

### 3.4. Data Analysis

We first used descriptive statistics to present the distribution of the different datasets and to describe the socio-demographic characteristics of the respondents. We calculated the median, mean, and standard deviation of the IDL depending on the following factors: *app category*, *time of day in hours*, *charging*, *battery level*, *sex* (only DS2 and DS3; *sex* refers to the binary sex assigned at birth), *age* (only DS2 and DS3), and *Big Five personality traits* (only DS3). Regarding *time of day in hours*, for all analyses, we truncated all local time values after the specification of the hour. In other words, we only considered the number of hours and did not round.

We then conducted a bivariate analysis of the data using a compared mean test to compare the mean of the IDL of different groups. More specifically, we determined whether the associated means of the various specifications of one variable were significantly different from each other. Thereby, we applied different kinds of tests depending on the characteristic of the variable. For categorical variables, such as the Sex or App category, we used a *t*-test, if the variable has only two categories (e.g., charging), or ANOVA (analysis of variance), if the variables has more than two categories (e.g., app category). For continuous variables, such as battery level, the Spearman correlation was applied. To measure the significance, we chose a significance level of 5%. In the subsequent multiple linear regression model, all variables for which the p-value was less than 0.05 in the bivariate analysis, were included to model the linear relationship between those explanatory variables and the IDL. The model predicts the IDL based on the values of the explanatory variables. Before the regression was conducted, the important requirements for this analysis, such as the absence of multicollinearity, have also been reviewed and confirmed. In order to include the categorical variables in the linear regression model as well, they had to be transformed into dummy variables. A dummy variable is a binary variable that can take only the values 0 or 1, representing the observation of a characteristic (e.g., being male was equal to 1 or not being male was equal to 0). For each categorical variable, which can take on *k* different values, k−1 dummy variables were included in the regression model to avoid perfect collinearity. Thus, the specifications *not charging*, *Messaging*, as well as *female* are used as reference categories within the individual features. All analyses were two-sided with a significance level of p<0.05.

## 4. Results

The characteristics of the users and the distribution of interaction delays in minutes are summarized in Table 3. DS1 included 9,894,656 notifications from 922 users, DS2 included 5,794,439 notifications from 537 users and included factors such as *sex* and *age*, and DS3 included 4,396,241 notifications from 417 users and included the variables from DS2 as well as the *Big Five personality traits*.

Regarding the optimizations and limitations introduced in Android 6 and 7 (see Section 2.2), we analyzed the Android version of the devices. Over 70% of the dataset was collected from devices running Android 8, followed by Android 9 and Android 7 as the second and third most common versions, respectively. The remaining data accounted for less than 0.2% of DS1.

When examining the apps that trigger notifications, it is evident that the quantity of records gathered differs significantly among the app categories (see Table 3). More than half of the data can be attributed to the *Messaging* group, which comprises both messenger and email apps (e.g., *Telegram Messenger* and *Google Mail*), accounting for a substantial portion of the records in DS1. According to Table 4, the messenger app *WhatsApp* on its own accounts for 35% of DS1.

Table 3 displays both the mean and median IDL for the apps in our dataset. The mean is the arithmetic average and represents the central tendency of the data. However, since outliers (i.e., notifications not responded to for a long time) are not uncommon in mobile crowdsensing studies, we also calculate the median to provide an additional measure for data understandability. The median represents the middle value of an ordered dataset (i.e., the IDL of 50% of all notifications is lower). Therefore, when comparing the median IDL of two app categories, we obtain a less biased view of the data.

The average IDL of *WhatsApp* (mean = 4.71; median = 0.23) is relatively low compared to other frequently used apps. It is important to note that the *Messaging* app category includes apps associated with social networks, such as *Facebook Messenger*. The distinction between social media apps and messenger apps is based on their main function, such as instant messaging, rather than their affiliation, such as *Facebook*.

Although the median for similar apps in the *Messaging* app category, such as *Telegram* (package name: com.telegram.messenger) or *Facebook Messenger* (package name: com.facebook.orca), is comparatively low, their means differ significantly. Email applications such as *Google Mail* (package name: com.google.android.gm) and Microsoft’s email app *Outlook* (package name: com.microsoft.office.outlook) exhibit significantly higher mean values.

The category with the second-largest number of apps is *System_OS*, with over 2 million records (23.5%). For instance, Android’s download manager is part of this group, ranking third among the top 10 apps in DS1. Following the two largest app categories, there is a significant decrease in notification frequency: the third-largest group, *News_Entertainment*, accounts for only 5%, and all other groups each have a share of less than 5% (see Table 3).

**Figure 1:**
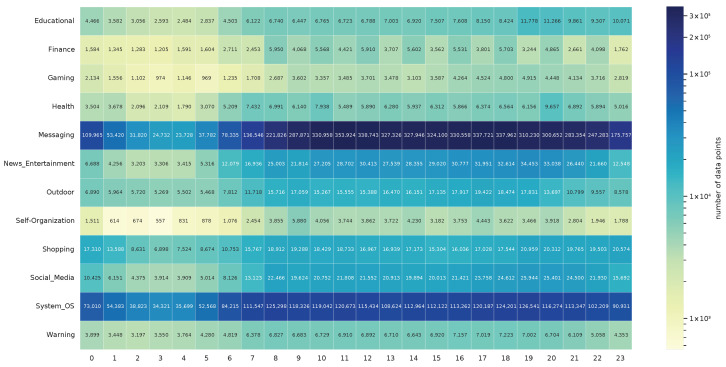
Records of each app category in DS1 per time of day in hours.

Figure 1 also illustrates the large differences in the number of records per group. It shows the number of notifications for each hour of the day and app category. Blue cells indicate a large number of records, yellow cells indicate only a few data, and green cells indicate everything in between. When comparing the app categories *Messaging* and *Gaming*, *Messaging* showed 66 times more data usage per hour between 10 a.m. and 8 p.m. Large differences are also noticeable in less populated categories such as *Entertainment* and *Self_Organization*. The dataset also indicates that fewer notifications are generated at night due to reduced interaction with the smartphone during these times. For instance, in the *Messaging* app category, the number of records increases thirteen-fold between 3 a.m. and 6 p.m, while categories like *Warning* only show a minor increase during the same period.

**Figure 2:**
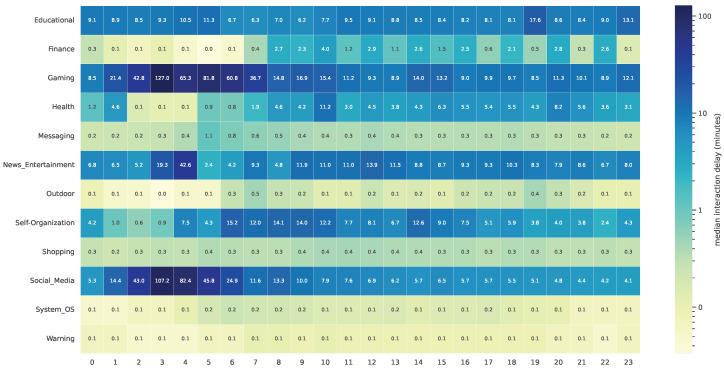
Median IDL of each app category in DS1 per time of day in hours.

The median IDL also varies greatly depending on the app category. Figure 2 displays the distribution of the IDL throughout the day and per app category. While some categories exhibit less variation across the day (e.g., *Educational* and *Messaging*), there is significant variation in categories such as *Gaming* and *Social_Media*. An increase in IDL often occurs at night, particularly between 1 and 6 a.m., with a peak at 3 or 4 a.m. Figure 2 also shows an opposing trend for some app categories, such as *Finance* or *Health*.

**Figure 3:**
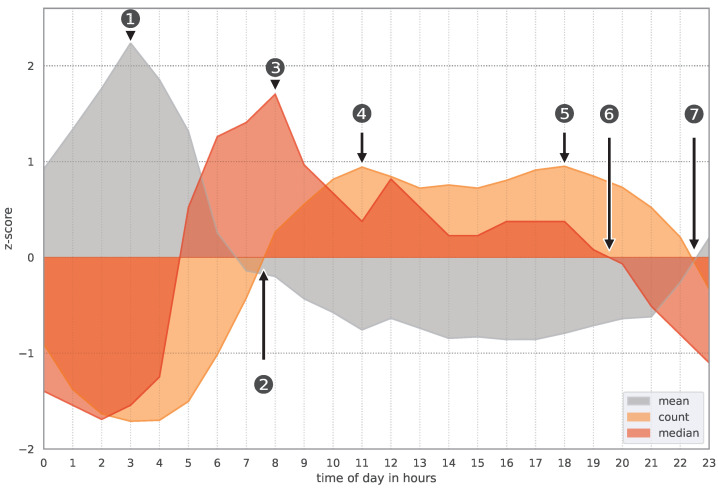
Z-scores for the mean IDL (gray), median IDL (red), and the record count (orange) per time of day in hours in DS1.

**Figure 4:**
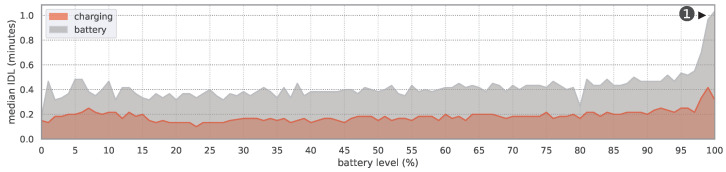
Median IDL for each battery level during charging and battery operation in DS1 (non-stacked areas).

**Figure 5:**
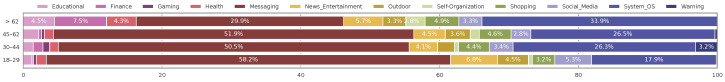
Percentage of app categories per age group in DS2.

To investigate the association between the mean and median IDL and the record count, we calculated their z-scores. This allows for the comparison of different measures and a better observation of fluctuations over time, as the z-scores indicate the difference between a value and the mean in terms of standard deviations. The z-scores were grouped by time of day in hours and are shown in Figure 3. This calculation enabled us to identify peaks throughout the day, indicating times with above-average values and potentially high IDL for new notifications, as well as periods with average or below-average IDL. The mean and median IDL show opposing trends. The lowest z-score for the median IDL is at 2 a.m., while the highest z-score for the mean IDL is at 3 a.m. (see Figure 3, ①). The third z-score for the record count indicates that the number of notifications is below average during these nighttimes. The number of notifications increases and is above average at around 7:30 a.m. (see Figure 3, ②). Although the median IDL also increases until 8 a.m. (see Figure 3, ③), the mean IDL decreases to a below-average value. After 8 p.m., the median IDL decreases to an average value while the notification count increases until 11 a.m. (see Figure 3, ④) and remains a relatively stable above-average value until 6 p.m. (see Figure 3, ⑤). Between 7:30 p.m. and 10:30 p.m., the median IDL and record count are below average, making it a promising time for user notifications.

The bivariate analysis (see Table 3) revealed a significant difference in the IDL among various app categories (*p*-value < 0.001). The smallest IDL was observed for *Warning* notifications (mean = 2.71; median = 0.07), while the highest IDL was observed for *Gaming* notifications with a median of 12.75 (mean = 64.1). The median interaction delays for the *Messaging*, *Outdoor*, *Shopping*, and *System_OS* app categories were all less than one minute.

The Spearman correlation indicates a significant association between the *time of day in hours* and the IDL, as well as between the *battery level* and the IDL (see Table 3). Although the number of notifications between 12 and 5 a.m. was relatively small, the median IDL during this period was the lowest, averaging 0.18 min. When examining the battery level, it is evident that the IDL decreases as the battery level decreases. The mean IDL for low battery levels is 13.41 with a median of 0.27, while the mean IDL for high battery levels is 19.53 with a median of 0.40. Most of the time, the smartphones were not charging (75%). However, the device’s charging status is significantly associated with the IDL. When the device is charging, the IDL tends to be smaller (mean = 14.93; median = 0.2) compared to when it is not charging (mean = 17.04; median = 0.42). Figure 4 (red: charging = yes; gray: charging = no) illustrates the difference between the two charging states, showing a steady distance between the two median values for the IDL for each battery level.

All significant features from the bivariate analyses were included in the subsequent multiple linear regression. The results, presented in Table 5, confirm the previous findings that a higher battery level is associated with a higher IDL, while the IDL is smaller later in the day. On average, the interaction delay decreases by 0.43 min for every additional hour during the day. Additionally, an increase of 1% in battery results in an average delay increase of 0.1 min. On average, the IDL differs depending on whether the smartphone is charging or not by 2.8 min. Additionally, the app category is correlated with the IDL value. For instance, notifications categorized as *Warning* have an IDL that is 8.9 min less than those categorized as *Messaging*. The IDL of all other categories is higher compared to messaging.

### 4.1. The Role of Sex and Age

For DS2, we recalculated the z-scores for the mean and median IDL, as well as for the notification count. The distribution and results were comparable to those of DS1. Therefore, the bivariate analyses of the factors already included in DS1 remained significant. The distributions within the features did not differ much from those in DS1. In addition to the analyses of DS1, we also included the association of *sex* and *age* with the IDL. Both the t-test for *sex* and the Spearman correlation for *age* were significant, indicating that there are statistically significant differences between their IDL means.

**Figure 6:**
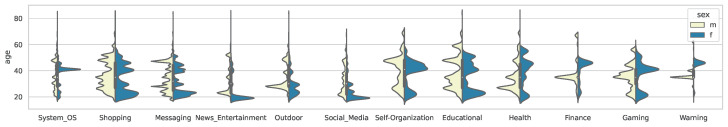
Number of notifications of female and male users for each app category in DS2.

Since *sex* and *age* had a significant effect on IDL, we examined the app categories for deviations. Table 6, illustrates the findings for both sexes. Most app categories have comparable IDLs for female and male users with only minor differences. However, the same app categories (e.g., *News_Entertainment*, *Finance*, and *Gaming*) showed considerable deviations, which is due to the heterogeneous distribution of the data (see Table 6, SD). We also investigated in the number of notifications per app category depending on *age* (see Figure 5 and Figure 6). It was found that younger individuals, particularly those aged 18 to 29, receive a higher number of notifications for the app categories *Messaging* and *Social_Media* compared to older age groups. Additionally, the number of notifications in the *News_Entertainment* category is also higher among younger individuals than in other age groups. All age groups have similar percentages of applications for communication, as shown in Figure 5. Some deviations, such as the noticeably higher number of notifications in the categories of *Finance* and *Health*, are particularly evident among individuals aged over 62.

**Figure 7:**
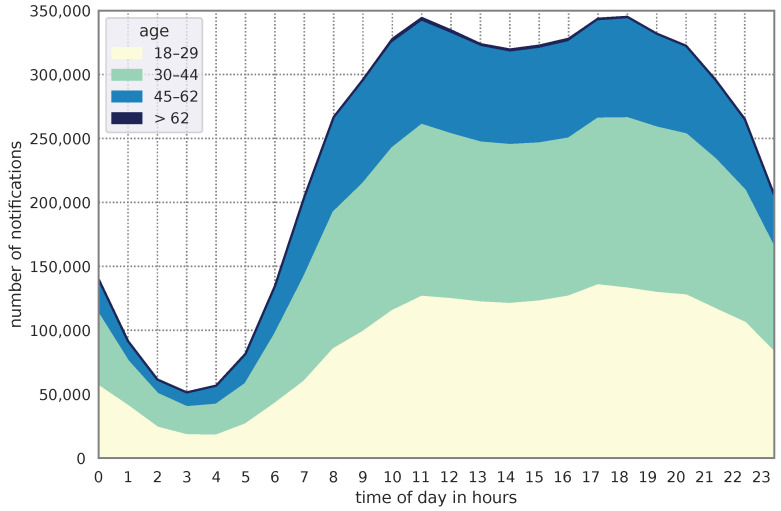
Number of notifications of each age group throughout the day in DS2 (stacked areas).

Table 3 shows the differences between male and female users for the entire dataset. Female users have a longer median reaction time to notifications (median = 0.43) compared to male users (median = 0.33). Additionally, the average interaction delay increases with age. In DS2, the mean IDL for the 18–29 age group and the 45–62 age group differs by approximately 9 min. The median time differs slightly, with 0.30 min for the 18–29 age group and 0.55 minutes for the 45–62 age group. The data indicate that 37.2% of the sample belonged to the 18–29 age group, while 23.0% belonged to the 45–62 age group. Furthermore, Figure 7 illustrates that the distribution of notifications during the day was similar across all age groups. Additionally, the data reveal that individuals under the age of 45 receive more notifications at night. Especially for the age group of 45–62 years, there is a clear decrease in the number of notifications at 10 p.m.

**Figure 8:**
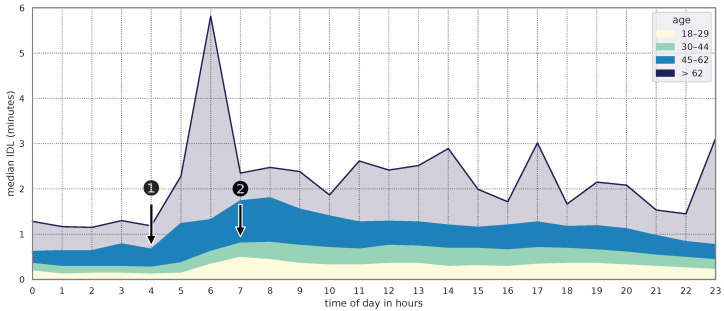
Median interaction delay (IDL) of each age group throughout the day in DS2 (stacked areas). Data of users older than 62 are presented transparently because only little data were available (0.7% of DS2).

Figure 8 shows the median IDL in minutes for each age group throughout the day. The age groups of 18–29 years and 30–44 years exhibited few differences, with the exception of a peak at 7 a.m. in the 18–29 age group (see Figure 8, ②). Additionally, the median IDL in the 18-29 age group only increases from 5 a.m. onward, in contrast to all other age groups (see Figure 8, ①). Users in the 45–62 age group show an increased median during nighttime and especially in the morning compared to younger users. Figure 8 also showed multiple peaks for users above 62 years, having the largest peak at 6 a.m. Since we have only little data for this age group, only 0.7% of DS2 to be precise (see Table 3), these peaks represent the outliers of individual users rather than being representative. Therefore, we have depicted the area of users over 62 years old transparently. As all features, including *age* and *sex*, were significantly associated with IDL in this dataset, they were all included as explanatory variables in the multiple linear regression. The regression results are comparable to those of DS1. The battery level and time of day values are consistent with the regression model for DS1. However, the charging value differs slightly (−3.87 compared to −2.80), while the values of the different app categories deviate significantly from those in DS1 (e.g., *Finance*: DS1: 20.34, DS2: 8.74). In addition to the previously mentioned variables, the newly added factors of *age* and *sex* are also significantly associated with the IDL. On average, a person who is one year older requires 0.39 more minutes to react to a notification. Additionally, male users require more time to react than female users.

### 4.2. The Role of the Big Five Personality Traits

DS3 showed similar z-scores for the mean and median IDL, as well as a comparable notification distribution throughout the day when compared to DS2. The Big Five personality trait scores were also included in DS3, in addition to the factors included in DS2. In the bivariate analysis, all Big Five personality traits (*Openness*, *Conscientiousness*, *Extraversion*, *Agreeableness*, and *Neuroticism*) are significantly and positively related to the IDL. This means that a higher score in these individual personality traits is associated with a higher IDL. To examine the connection between these factors and the IDL, we used the Spearman correlation. The measure resulted in a *p*-value < 0.001 for all five personality traits, indicating statistical significance.

Individuals with higher scores in *openness*, *conscientiousness*, and *neuroticism* tend to have less interaction delay compared to those with lower scores. For instance, those in the top half of openness score have a median interaction delay of 0.28, while those in the bottom half have a median interaction delay of 0.4. The median of both halves of the extraversion score is identical. However, individuals with a score in the upper half of agreeableness exhibit a higher interaction delay than those in the bottom half (median = 0.45 compared to 0.25).

The multivariate regression model, which includes the Big Five personality features, confirms the results of the bivariate analysis. All newly added variables, except for *extraversion*, are significantly associated with IDL. On average, a one-point increase on the *neuroticism* scale is linked to a 1.44 higher IDL, whereas a one-point increase in *openness*, *conscientiousness*, and *agreeableness* raises the IDL by less than half a minute.

## 5. Discussion

The analysis of the TYDR dataset showed that there is a significant dependency between the IDL and the app category. In the case of the category *Gaming*, for example, the difference in the mean IDL compared to the category *Warning* was over an hour (63.36 min). A deviation of interaction times between app categories was also found in the works [4,14,15]. In all works, the app category *Messaging* (called “Messenger” in [4,15] and “SMS & IM” in [14]) also showed a lower interaction delay than the other categories. This difference in behavior may indicate that users saw the notifications and intentionally did not respond to them. Especially if users prioritize notification differently depending on the app category. In [4], users are asked to give feedback on notifications and also to prioritize them. It turns out that messenger apps were given the highest priority in this study. Moreover, the interaction time (in [4] called “click time”) and the importance of notifications showed a negative correlation and, therefore, confirms the results of our analysis with regard to the app category.

The distribution of app categories is also similar to works [4,14,15]: the category of messaging apps contains the most notification data. However, our work stands out from other works because we extracted the number and difference in notifications per time of day more precisely than, for example, ref. [15]. Moreover, we identified in all three datasets that the IDL is above average in the morning hours (see Figure 3, ③). A similar trend is shown in a study of [25]: they found that highest number of notifications per hour are posted in the morning, and the lowest at night and in the evening. Interestingly, their application allowed users to snooze notifications and the highest number of snoozes per hour was also in the morning. We therefore propose to schedule notifications in the evening, since our data showed that both the amount of notifications and the median IDL is decreasing after 8 p.m. (see Figure 3, ⑥,⑦). The results of the linear regression support this finding, as IDL is negatively correlated with time of day and decreases throughout the day.

Using this insight, apps that require a short IDL (e.g., therapy apps that require an action within a short time frame [9]) can optimize their notification scheduling. Especially, when apps are using an alarm-based approach (see Section 2.2) to implement local push notifications. This approach is generally more robust than remote push notifications, because of the network restrictions that are part of the battery optimizations of current Android operating system versions [36].

To further support the scheduling of future notifications, we calculated the z-scores for the mean and median IDL as well as for the notification count. This supported our understanding of the average IDL per hour. More precisely, using the mean z-score, we were able to identify points in time with, on average, high or low IDL (see Figure 3, high: ①; low: between ② and ⑦). The same is the case for the median z-scores, which offered us a better view on the average IDL with less dispersion due to outliers. Time points with high median z-scores but only average mean z-scores (i.e., at these time points the IDL was increased above average and this was not due to outliers) show intervals at which no notification should be sent (see Figure 3, ③). Combined with the third z-score for the record count (i.e., the average amount of notifications), we could identify a time span with low mean and median z-scores and thereby decreasing notification count (see Figure 3, between ⑥ and ⑦). This indicates that the users were active and reacted very quickly at these points in time. Furthermore, because of the decreasing notification count, users are maybe more likely to perceive new notifications.

Looking at Table 5 and Figure 4, the charging state is also linked to the IDL. In Figure 4, the continuous distance visualizes the existence of an influencing factor with respect to the IDL. In case of the charging state, there are technical as well as behavioral reasons for this. On the technical side, the absence of battery optimizations (as described in Section 2.2) has a positive effect on the background execution of apps. Background services do not have to wait for a maintenance window to receive and create push notifications. Another possible reason could be the user’s behavior during the charging process. If the user charges their smartphone at daytime or during their waking phase, the probability that the user will also use the smartphone is high. Interestingly, in Figure 4 ①, an increased IDL difference is shown between 98% and 100% battery level. This difference might be caused by the fact that after charging the smartphone, a user starts another activity (e.g., going to work). This possible activity would match the increased IDL in the morning shown in Figure 8. It is also possible that the user is more likely to charge the smartphone next to him/her or look at the smartphone more often in order not to miss a notification. Because the OS does not restrict any app’s request for resources during charging, an accumulation of interruptions caused by notifications (apps no longer have to wait for the next maintenance window to create or receive notifications) is also a possible reason for a lower IDL (see Section 2.2).

Demographic information about the user, especially age, was significantly associated with the IDL. A comparable association between age and the notification interaction is described in [26], where the authors developed an algorithm using machine learning techniques to predict the likelihood of user interaction with a smartphone notification. For their computation, they used a sample with similar age distribution (mean = 37.85; std = 11.01) to our sample (DS2: mean = 35.04; std = 10.67). In contrast to their work, we did not compute the likelihood of a user interaction, but tried to predict the difference in time between displaying a notification in the notification bar and its removal. Consequently, we also identified age as an important factor, but did not reproduce the positive correlation between age and user interaction because all notifications without interaction were removed from our dataset during data cleaning (see Section 3.2).

Another demographic information, the user’s sex, is associated with the IDL. Females tend to have a slightly larger median IDL compared to males. On the other hand, the mean IDL is slightly larger compared to males. These differences might be due to varying levels of IDL within sex groups, as females show fewer outliers with respect to IDL. Another possible reason is the unequal distribution of data between men and women (see Table 3). Since according to [23], daily smartphone use for males is lower compared to females, this difference in smartphone use could lead to a lower average IDL for females.

When we had a closer look at the Big Five personality traits, the regression analysis showed that people who have a higher neuroticism score also have a higher IDL on average. Neurotic people are defined as rather emotionally unstable, impulsive people, who tend to get angry fast and perceive life negatively [37]. Other studies have found that neuroticism is significantly positively related to higher smartphone [22] and social media usage [38] or even to a smartphone addiction [39,40]. While more neurotic users use their phone more, their IDL was higher. Even if someone has a higher smartphone usage, he/she does not necessarily respond to notifications more quickly.

### 5.1. Implications for Notification Scheduling

The objective was to utilize minimal data, commonly available in most apps, to identify patterns in the user’s smartphone interaction and facilitate intelligent notification planning. These findings can be directly applied to the creation of intelligent notification systems. Intelligent notification systems can utilize our results to calculate the expected IDL. We would like to point out that the parameters for the planning algorithms are available at different times. The app category, notification volume, and demographic data, such as gender and age as well as the Big Five personality traits are examples of data that are known prior to operation. They are suitable for calculating and defining individual tendencies for notifications a priori. On the other hand, there are parameters that are only available to the notification system during operation and are therefore suitable for ad hoc calculations. These include, for example, the battery level or the state of charge. The time of day is a parameter that can be used both for a priori calculations (e.g., to determine time periods) and for ad hoc calculations.

The study showed that the number of notifications and the IDL are significantly affected by the app category and time of day. Scheduled notifications for apps from categories that are expected to have a higher IDL, according to our analysis, can use the regression results to choose a more appropriate time to send notifications to the user, thereby reducing the IDL. For example, our analysis indicates to schedule notifications in the evening, since our data showed that both the amount of notifications and the median IDL decreases after 8 p.m.

Furthermore, the IDL is significantly influenced by the user’s age, gender, charging state, and current battery level. Health apps can use user demographic data to personalize notifications. For instance, older individuals, who typically have a higher IDL, could receive scheduled notifications earlier to perform actions, such as data entry, within a certain time interval. This is especially important in studies that use daily life methods, when a person’s momentary state is to be recorded in a short time interval [9]. The same applies to gender, as men tend to have a higher IDL. To support the development of intelligent notification systems, developers are also encouraged to monitor the current battery level and state of charge and integrate them into their calculations.

### 5.2. Limitations

In this work, the time between creation and removal of a notification from the notification bar was measured to reflect user response time. As discussed earlier, users prioritize notifications differently, suggesting an impact on IDL. Since this study used data from a mobile crowdsensing app, real-world measurements were used for evaluation without including the participants’ prioritization. The participants of the TYDR study (i.e., app users) were not instructed to respond as quickly as possible, but rather real-world behavior was measured. This results in a high external validity (a strength of mobile crowdsensing research [5]), but at the cost of the internal validity. In other words, we cannot differentiate whether a high IDL is caused by technical issues or the app user’s individual prioritization of apps without the collection of more data (e.g., screen activation). To achieve this, there are specialized tools to research both app and user behavior [10].

Another limitation of this study is the lack of more notification metadata in the TYDR dataset. In newer Android versions, it is possible to get the reason of the notification removal from the OS via the notification listener (see Section 3.1). This field returns an encoded value with the information if, for example, the user or the app itself removed the notification from the notification bar [41]. This information is important to further clean the dataset and especially to implement an improved detection mechanism for permanent notifications or progress indicators. In the present work, this information was not available, so we could only process the data by making assumptions (see Section 3.2). In addition, this would allow more detailed analytics on how the user interacts with the notifications (e.g., click and dismiss rates for notifications per app category).

Thus, there is more dispersion in the data, which can be seen in Table 3 by the difference between the arithmetic mean and the median as well as the standard deviation of the IDL. In addition, knowing whether a notification was triggered locally or remotely would help us better understand the impact of battery optimizations in Android smartphones. This information could be used to improve the notification systems for services with a need for a short IDL. Furthermore, in contrast to [27], we did not differentiate between individual and group messages in our analysis of the app category *Messenger*.

Additionally, because we used real-world data, the number of the individual values a variable can take on, diverges. For example, considerably more male than female users are included in this analysis and, in contrast to the number of messaging notifications, the number of *Finance* or *Gaming* notifications is rather small. We partially addressed this issue by only including categories with a sufficient number of notifications and/or enough unique users (more than 10, see Section 3.2).

The same applies with the age distribution of the users. The majority of notifications was collected from users younger than 62 years. Only 0.7% of notifications in DS2 and 1.0% of notifications in DS3 can be assigned to users older than 62 years (see Table 3). In Figure 7, the distribution of the data over the day is shown. Notifications from users older than 62 years are colored in dark blue. Because these notifications are only a fraction of the entire dataset, the gained information (e.g., IDL of age group > 62 in Figure 8) must be interpreted with caution. The influence of individual outliers is much more pronounced with a small user base, so that the significance of the data is reduced.

In addition, we did not include users’ geographic or cultural origin in the analysis in this study. The TYDR app was available worldwide on the Google Play Store and, therefore, any cultural differences may be part of the dataset. An analysis of notifications for such differences is part of future work.

Finally, we would like to note that this study only includes data from users with an Android smartphone. Since interaction with operating systems may differ in terms of notifications (e.g., due to different implementations of battery optimization features), the results are not directly applicable to users of other operating systems, such as Apple’s iOS.

## 6. Conclusions

In this work, we presented a detailed study on smartphone notifications to identify influencing factors on the interaction delay. The used data are part of the TYDR dataset and contains almost 10 million notifications that were collected in-the-wild from 922 unique users.

We found that the number of notification is significantly depending on the app category and the time of the day. For example, the number of notifications created by the app *WhatsApp* on its own accounts for 35% of the data, and showed a thirteen-fold increase between 3 a.m. and 6 p.m. Furthermore, our comprehensive analysis of the temporal difference between notification creation and removal in the notification bar showed a significant negative association between the interaction delay and the time of day. In other words, response time decreases over the course of the day. We thus propose to, if possible, notify users in the evening between 8 and 11 p.m.

We used additional data provided by either the users (e.g., sex and age) or the device (e.g, battery level) to identify more factors that possibly influence the interaction delay. Our analysis showed a significant positive association between the interaction delay and the battery level as well as age. In other words, younger users tend to interact faster, and app users on fully charged devices exhibit a higher IDL than on devices with lower battery levels. We also found that males tend to have higher interaction delays than females.

Since the majority of participants in our study were under the age of 62, and the results for those over 62 were based on a smaller sample size, it would be beneficial to conduct further studies, particularly with older participants. Additionally, it is possible that other smartphone parameters provided to developers may impact user interaction. The incorporation of the latter into the development of smart notification systems could assist in tailoring the planning and ad hoc scheduling of survey and notification periods to the user. This is particularly relevant in the case of ecological momentary assessment or experience sampling apps.

We highly encourage researchers and developers to incorporate more information about the user and the smartphone in their notification scheduling algorithms, since additional factors like the Big Five personality traits or the devices’ charging state also correlated with the interaction delay. In the pursuit for strategic notification planning, our contribution can help to identify opportune moments for future user notifications.

## Figures and Tables

**Table 1:** Comparison of the parameters of the analysis between the related work and the study presented in this paper.

Study	# Users	# Records	Demographic Information	Personality Traits	Notification Interaction	App Metadata	Battery Level
Exler et al. [1]	23	N/A	No	Extraversion	No	No	No
Sahami et al. [4]	40,000	200,000,000	No	No	Response Time,	App Category,	No
					Notification Volume,	Package Name	
					Notification Importance		
Pielot et al. [11]	278	794,525	No	No	Response Time,	Package Name	No
					Notification Volume		
					Notification Category		
Weber et al. [14]	3953	>8,000,000	No	No	Notification Volume,	App Category,	No
					Notification Drawer Position,	Package Name	
					Notification Priority,		
					User Types		
Pielot et al. [15]	15	6854	No	No	Response Time,	Package Name	No
					Notification Volume,		
					Notification Category		
Chen et al. [22]	869	794,525	Sex	Extraversion,	No	No	No
				Neuroticism,			
				Psychoticism			
Mehrotra et al. [27]	20	10,372	No	Openness,	Interaction Rate,	No	No
				Conscientiousness,	Response Time,		
				Extraversion,	Notification Priority,		
				Agreeableness,	Alert Modality		
				Neuroticism,			
Weber et al. [25]	295	20,345,277	No	No	Notification Volume	App Category,	No
						Package Name	
Pielot et al. [26]	337	794,525	Age	No	Engagement		Yes
Andone et al. [23]	30,677	794,525	Sex,	No	No	App Category	Yes
			Age				
Our Study	922	9,894,656	Sex	Openness,	Response Time,	App Category,	Yes
			Age	Conscientiousness,	Notification Volume	Package Name	
				Extraversion,			
				Agreeableness,			
				Neuroticism,			

**Table 2:** Overview of the used datasets. DS1 contains only device- and app-related data. DS2 contains filled out demographic questionnaires. In DS3, users also filled out an additional personality questionnaire. Therefore, DS3 is a subset of DS2 and DS2 is a subset of DS1. Percentages have been rounded.

	DS1	DS2 ^1^	DS3 ^2^
Sample Size	9,894,656 (100%)	5,794,43 (58%)	4,396,241 (44%)
Unique Users	922 (100%)	537 (58%)	417 (45%)
Females	-	86 (16%)	82 (20%)
Males	-	451 (84%)	335 (80%)

^1^ DS includes a demographic questionnaire. ^2^ DS includes both the demographic questionnaire and a Big Five personality traits questionnaire.

**Table 3:** Descriptive statistical analysis of DS1, DS2, and DS3. Demographic information is only available in DS2 and DS3. Data on Big Five personality traits are only available in DS3.

	DS1 (*n* = 922)					DS2 (*n* = 537)					DS3 (*n* = 417)				
	*n* (%)	Median	Mean	SD	*p*-Value	*n* (%)	Median	Mean	SD	*p*-Value	*n* (%)	Median	Mean	SD	*p*-Value
	9,894,656 (100%)	0.35	16.52	67.61		5,794,439 (100%)	0.35	15.37	63.17		4,396,241 (100%)	0.33	15.03	60.48	
**Sex**															
Female	NI					680,196 (11.7%)	0.43	12.78	52.15	<0.001 ^^^	661,565 (15.0%)	0.43	12.72	51.92	<0.001 ^^^
Male						5,114,243 (88.3%)	0.33	15.71	64.49		3,734,676 (85.0%)	0.32	15.44	61.87	
**Age groups**															
18–29						2,153,202 (37.2%)	0.30	11.09	47.19	<0.001 *	1,717,147 (39.1%)	0.28	10.56	44.79	<0.001 *
30–44	NI					2,263,726 (39.1%)	0.32	16.18	67.32		1,686,218 (38.4%)	0.30	16.98	67.95	
45–62						1,335,274 (23.0%)	0.55	20.38	75.75		950,735 (21.6%)	0.52	18.92	68.51	
>62						42,237 (0.7%)	0.83	31.43	87.43		42,141 (1.0%)	0.85	31.50	87.52	
**App category**															
Educational	161,001 (02%)	8.98	28.63	75.13	<0.001 ^^^	91,312 (1.6%)	8.62	26.16	68.29	<0.001 ^^^	75,768 (1.7%)	8.83	25.51	64.51	<0.001 ^^^
Finance	84,229 (01%)	1.35	31.43	96.13		45,889 (0.8%)	0.10	20.17	77.40		42,453 (1.0%)	0.08	18.64	75.11	
Gaming	71,444 (01%)	12.75	64.1	129.03		35,545 (0.6%)	13.77	65.49	129.64		26,979 (0.6%)	13.82	64.77	117.65	
Health	132,284 (01%)	3.90	39.38	108.72		77,868 (1.3%)	4.37	38.59	100.40		67,054 (1.5%)	4.28	36.83	92.65	
Messaging	5,332,539 (54%)	0.33	10.49	45.32		3,103,692 (53.6%)	0.32	9.62	41.79		2,341,035 (53.3%)	0.33	10.11	42.70	
News-Entertainment	496,731 (05%)	9.30	55.44	136.8		301,532 (5.2%)	9.92	51.18	130.74		155,005 (3.5%)	9.38	60.07	137.74	
Outdoor	299,359 (03%)	0.17	16.97	61.69		201,299 (3.5%)	0.12	14.86	55.18		178,017 (4.0%)	0.10	12.68	47.95	
Self-Organization	66,866 (01%)	7.30	46.07	109.5		44,405 (0.8%)	6.85	46.21	108.61		32,707 (0.7%)	5.00	44.45	109.60	
Shopping	382,611 (04%)	0.33	22.99	88.85		231,964 (4.0%)	0.32	22.51	85.21		174,207 (4.0%)	0.35	21.74	80.86	
SocialMedia	405,317 (04%)	6.88	51.09	125.36		232,144 (4.0%)	5.12	42.65	104.39		176,853 (4.0%)	5.02	40.30	98.25	
System OS	2,324,001 (23%)	0.13	10.72	55.92		1,348,839 (23.3%)	0.15	10.98	55.77		1,046,461 (23.8%)	0.13	10.91	53.91	
Warning	138,274 (01%)	0.07	2.71	20.18		79,950 (1.4%)	0.07	1.75	16.22		79,702 (1.8%)	0.07	1.64	15.69	
**Time of day in hours**															
12–5 a.m.	806,622 (08%)	0.18	31.29	102.2	<0.001 *	482,604 (8.3%)	0.18	28.86	94.66	<0.001 *	385,514 (8.8%)	0.17	28.19	91.94	<0.001 *
6–11 a.m.	2,688,363 (27%)	0.43	16.71	63.3		1,573,411 (27.2%)	0.43	15.92	60.04		1,163,349 (26.5%)	0.40	15.58	55.87	
12–5 p.m.	3,372,461 (34%)	0.38	13.56	56.08		1,972,049 (34.0%)	0.38	12.43	52.08		1,508,429 (34.3%)	0.37	11.90	48.97	
6–11 p.m.	3,027,210 (31%)	0.30	15.71	70.89		1,766,375 (30.5%)	0.32	14.47	65.91		1,338,949 (30.5%)	0.30	14.29	64.05	
**Charging**															
no	7,452,097 (75%)	0.42	17.04	68.37	<0.001 ^^^	4,343,592 (75.0%)	0.42	16.00	64.34	<0.001 ^^^	3,288,858 (74.8%)	0.42	15.69	61.48	<0.001 ^^^
yes	2,442,559 (25%)	0.20	14.93	65.19		1,450,847 (25.0%)	0.20	13.48	59.51		1,107,383 (25.2%)	0.18	13.10	57.39	
**Battery level**															
Low (0–30%)	1,340,702 (14%)	0.27	13.41	58.81	<0.001 *	829,101 (14.3%)	0.27	12.32	55.78	<0.001 *	633,107 (14.4%)	0.25	12.06	53.55	<0.001 *
Middle (31–70%)	3,924,718 (40%)	0.33	14.03	59.19		2,387,027 (41.2%)	0.35	13.49	57.71		1,820,266 (41.4%)	0.33	12.79	53.74	
High (71–100%)	4,629,236 (47%)	0.40	19.53	76.03		2,578,311 (44.5%)	0.38	18.09	69.80		1,942,868 (44.2%)	0.38	18.10	67.97	
**BFI**															
Openness	NI					NI									
<3.0											640,252 (14.6%)	0.43	14.82	59.95	<0.001 *
>3.0											3,755,989 (85.4%)	0.32	15.07	60.57	
Conscientiousness	NI					NI									
<3.0											1,017,425 (23.1%)	0.3	13.72	56.93	<0.001 *
>3.0											3,378,816 (76.9%)	0.35	15.43	61.51	
Extraversion	NI					NI									
<3.0											1,466,851 (33.4%)	0.33	16.5	66.76	<0.001 *
>3.0											2,929,390 (66.6%)	0.33	14.3	57.07	
Agreeableness	NI					NI									
<3.0											471,728 (10.7%)	0.17	12.46	53.63	<0.001 *
>3.0											3,924,513 (89.3%)	0.37	15.34	61.25	
Neuroticism	NI					NI									
<3.0											3,188,353 (72.5%)	0.35	13.84	55.19	<0.001 *
>3.0											1,207,888 (27.5%)	0.32	18.18	72.53	
BFI															
Openness	NI					NI									
<3.82											2,053,224 (46.7%)	0.40	15.07	58.49	<0.001 *
>3.82											2,343,017 (53.3%)	0.28	15.00	62.17	
Conscientiousness	NI					NI									
<3.5											2,221,949 (50.5%)	0.40	15.28	61.55	<0.001 *
>3.5											2,174,292 (49.5%)	0.27	14.78	59.37	
Extraversion	NI					NI									
<3.35											2,136,688 (48.6%)	0.33	15.94	63.63	<0.001 *
>3.35											2,259,553 (51.4%)	0.33	14.17	57.34	
Agreeableness	NI					NI									
<3.67											2,164,723 (49.2%)	0.25	15.67	61.16	<0.001 *
>3.67											2,231,518 (50.8%)	0.45	14.41	59.81	
Neuroticism	NI					NI									
<2.74											1,809,642 (41.2%)	0.40	14.63	57.54	<0.001 *
>2.74											2,586,599 (58.8%)	0.30	15.31	62.45	

* Denotes *p*-value of Spearman’s correlation between continuous variable and interaction delay. ^^^ Denotes *p*-value of ANOVA or *t*-test for categorical variables. NI: Not included in data set.

**Table 4:** The top 10 apps with the most notifications in DS1 (IDL in minutes).

App Package Name	Frequency, *n* (%)	Mean	Median	SD
com.whatsapp ^1^	3,524,857 (35.62%)	4.68	0.23	23.99
com.google.android.gm ^1,2^	457,984 (4.63%)	21.40	0.85	67.42
com.android.providers.downloads	434,182 (4.37%)	0.87	0.02	17.04
com.android.vending	282,949 (2.86%)	11.89	0.23	67.54
org.telegram.messenger ^1^	280,649 (2.84%)	13.53	0.75	46.05
com.android.systemui	263,915 (2.67%)	11.13	0.22	61.59
com.facebook.orca ^1^	249,743 (2.52%)	10.21	0.22	50.05
com.samsung.android.incallui	175,156 (1.77%)	1.13	0.28	6.24
com.google.android.youtube	163,965 (1.66%)	38.12	5.88	109.27
com.microsoft.office.outlook ^1^	161,561 (1.63%)	41.90	9.55	99.85

^1^ Assigned to the group *Messaging*. ^2^ *Google Mail*.

**Table 5:** Results of the linear regression for DS1, DS2, and DS3.

	DS1				DS2				DS3			
	Beta	SE	*p*-Value	95% CI	Beta	SE	*p*-Value	95% CI	Beta	SE	*p*-Value	95% CI
**Constant**	11.03	0.09	<0.001	[10.86; 11.21]	−2.22	0.15	<0.001	[−2.51; −1.92]	−9.01	0.45	<0.001	[−9.90; −8.13]
**Battery level**	0.10	0.00	<0.001	[0.09; 0.10]	0.07	0.00	<0.001	[0.07; 0.08]	0.09	0.00	<0.001	[0.08; 0.09]
**Charging**												
No	Reference				Reference				Reference			
Yes	−2.80	0.05	<0.001	[−2.89; −2.70]	−3.87	0.06	<0.001	[−3.99; −3.75]	−3.88	0.07	<0.001	[−4.01; −3.75]
**Time of day in hours**	−0.43	0.00	<0.001	[−0.44; −0.42]	−0.41	0.01	<0.001	[−0.42; −0.41]	−0.41	0.01	<0.001	[−0.43; −0.41]
**App Category**												
Messaging	Reference				Reference				Reference			
Educational	17.90	0.17	<0.001	[17.57; 18.23]	14.79	0.21	<0.001	[14.39; 15.20]	13.33	0.22	<0.001	[12.90; 13.76]
Finance	20.34	0.23	<0.001	[19.89; 20.79]	8.74	0.29	<0.001	[8.17; 9.31]	6.09	0.29	<0.001	[5.52; 6.66]
Gaming	53.36	0.25	<0.001	[52.87; 53.85]	55.18	0.33	<0.001	[54.54; 55.83]	54.35	0.36	<0.001	[53.65; 55.06]
Health	28.21	0.18	<0.001	[27.85; 28.57]	28.00	0.22	<0.001	[27.56; 28.44]	25.62	0.23	<0.001	[25.16; 26.07]
News_Entertainment	44.79	0.10	<0.001	[44.59; 44.98]	41.95	0.12	<0.001	[41.72; 42.18]	49.67	0.16	<0.001	[49.37; 49.98]
Outdoor	5.87	0.12	<0.001	[5.63; 6.11]	4.31	0.14	<0.001	[4.03; 4.59]	1.24	0.15	<0.001	[0.95; 1.53]
Self-Organization	35.11	0.26	<0.001	[34.61; 35.62]	34.03	0.30	<0.001	[33.45; 34.61]	32.25	0.33	<0.001	[31.61; 32.90]
Shopping	12.66	0.11	<0.001	[12.44; 12.87]	12.46	0.13	<0.001	[12.20; 12.73]	11.34	0.15	<0.001	[11.05; 11.63]
Social_Media	40.22	0.11	<0.001	[40.01; 40.43]	33.82	0.13	<0.001	[33.56; 34.08]	30.81	0.15	<0.001	[30.53; 31.10]
System_OS	0.08	0.05	0.131 ^‡^	[−0.02; 0.18]	0.47	0.07	<0.001	[0.34; 0.60]	0.09	0.07	0.211 ^‡^	[−0.05; 0.23]
Warning	−8.90	0.18	<0.001	[−9.26; −8.55]	−9.48	0.22	<0.001	[−9.92; −9.05]	−10.73	0.22	<0.001	[−11.15; −10.31]
**Age**	NI				0.39	0.00	<0.001	[0.38; 0.39]	0.35	0.00	<0.001	[0.34; 0.35]
**Sex**	NI											
Female					Reference				Reference			
Male					0.58	0.08	<0.001	[0.43; 0.74]	1.83	0.09	<0.001	[1.67; 2.00]
**BFI**	NI				NI							
Openness									0.14	0.04	<0.001	[0.05; 0.22]
Conscientiousness									0.33	0.06	<0.001	[0.22; 0.44]
Extraversion									0.01	0.05	0.807 ^‡^	[−0.08; 0.11]
Agreeableness									0.38	0.06	<0.001	[0.26; 0.50]
Neuroticism									1.44	0.05	<0.001	[1.35; 1.54]

SE: Standard error. CI: Confidence Interval. NI: Not included in the regression model. ^‡^ Not significant.

**Table 6:** IDL in minutes per app category & sex in DS2.

App Category	Sex	IDL		
		**Median**	**Mean**	**SD**
Educational	Female	9.75	29.75	78.30
	Male	8.55	25.83	67.29
Finance	Female	23.87	81.82	126.82
	Male	0.08	17.63	73.57
Gaming	Female	0.52	22.03	63.92
	Male	16.37	70.36	134.15
Health	Female	5.87	45.40	100.29
	Male	4.20	37.85	100.39
Messaging	Female	0.38	9.06	37.97
	Male	0.32	9.70	42.30
News_Entertainment	Female	5.42	33.26	88.65
	Male	10.37	52.97	134.10
Outdoor	Female	2.98	19.61	53.72
	Male	0.12	14.74	55.21
Self-Organization	Female	2.34	40.23	94.75
	Male	7.52	46.98	110.24
Shopping	Female	0.30	20.72	84.07
	Male	0.33	22.70	85.33
Social_Media	Female	5.55	47.09	102.30
	Male	5.05	42.11	104.63
System_OS	Female	0.23	8.10	43.18
	Male	0.15	11.45	57.55
Warning	Female	0.05	2.94	10.75
	Male	0.07	1.73	16.29

## Data Availability

Data are contained within the article.
